# Integrating pharmacological evaluation and computational identification for deciphering the action mechanism of Yunpi-Huoxue-Sanjie formula alleviates diabetic cardiomyopathy

**DOI:** 10.3389/fphar.2022.957829

**Published:** 2022-09-05

**Authors:** Xin Zhang, Li-Yan You, Ze-Yu Zhang, Dong-Xiao Jiang, Yu Qiu, Ye-Ping Ruan, Zhu-Jun Mao

**Affiliations:** ^1^ School of Pharmaceutical Sciences, Zhejiang Chinese Medical University, Hangzhou, China; ^2^ Chinese Medicine Plant Essential Oil Zhejiang Engineering Research Center, Zhejiang, China

**Keywords:** YP-SJ formula, diabetic cardiomyopathy, traditional Chinese medicine, myocardial damage, FoxO1 signaling

## Abstract

**Background**: Yunpi-Huoxue-Sanjie (YP-SJ) formula is a Chinese herbal formula with unique advantages for the treatment of diabetic cardiovascular complications, such as Diabetic cardiomyopathy (DCM). However, potential targets and molecular mechanisms remain unclear. Therefore, our research was designed to evaluate rat myocardial morphology, fat metabolism and oxidative stress to verify myocardial protective effect of YP-SJ formula *in vivo*. And then to explore and validate its probable mechanism through network pharmacology and experiments *in vitro* and *in vivo*.

**Methods:** In this study, DCM rats were randomly divided into five groups: control group, model group, and three YP-SJ formula groups (low-dose, middle-dose, and high-dose groups). Experimental rats were treated with 6 g/kg/d, 12 g/kg/d and 24 g/kg/d YP-SJ formula by gavage for 10 weeks, respectively. Cardiac function of rats was measured by high-resolution small-animal imaging system. The cells were divided into control group, high glucose group, high glucose + control serum group, high glucose + dosed serum group, high glucose + NC-siRNA group, high glucose + siRNA-FoxO1 group. The extent of autophagy was measured by flow cytometry, immunofluorescence, and western blotting.

**Results:** It was found that YP-SJ formula could effectively improve cardiac systolic function in DCM rats. We identified 46 major candidate YP-SJ formula targets that are closely related to the progression of DCM. Enrichment analysis revealed key targets of YP-SJ formula related to environmental information processing, organic systems, and the metabolic occurrence of reactive oxygen species. Meanwhile, we verified that YP-SJ formula can increase the expression of forkhead box protein O1 (FoxO1), autophagy-related protein 7 (Atg7), Beclin 1, and light chain 3 (LC3), and decrease the expression of phosphorylated FoxO1 *in vitro* and *in vivo*. The results showed that YP-SJ formula could activate the FoxO1 signaling pathway associated with DCM rats. Further experiments showed that YP-SJ formula could improve cardiac function by regulating autophagy.

**Conclusion:** YP-SJ formula treats DCM by modulating targets that play a key role in autophagy, improving myocardial function through a multi-component, multi-level, multi-target, multi-pathway, and multi-mechanism approach.

## 1 Introduction

Diabetes is the most common metabolic disease in the world today, and morbidity and mortality are increasing year by year (Saeedi et al., 2019). Diabetic cardiomyopathy (DCM) is specific cardiomyopathy that occurs in diabetic patients, manifesting mainly as abnormal myocardial structure and function. It is independent of coronary heart disease, hypertension, valvular heart disease, and other heart diseases and is one of the main causes of death in diabetic patients (Gulsin et al., 2019). The occurrence of Type 2 Diabetic Mellitus (T2DM) is accompanied by myocardial damage, which causes myocardial diastolic dysfunction in asymptomatic diabetic patients, insulin resistance (IR), hyperglycemia, and hyperinsulinemia. T2DM also leads to myocardial cytotoxicity, microangiopathy, metabolic disorders, and oxidative stress, and contributes to the occurrence of DCM(Al Shamsi et al., 2006a; Al Shamsi et al., 2006b; Bersch-Ferreira et al., 2018; Zhang Y. et al., 2018; Rotariu et al., 2022). Hypoglycemia may increase the risk of cardiovascular events (Maack et al., 2018). Currently, there is no effective treatment for DCM.

Autophagy is the process of clearing cells of damaged organelles or debris. It can reduce the damage to cells or organelles caused by harmful metabolites and accelerate the decomposition of free fatty acids and advanced glycosylation products. Thereby improving diabetic myocardial ischemia and metabolic disorders in diabetes. Maintaining myocardial cell homeostasis and improving cardiac function in diabetes are of great significance (Chaanine, 2018). IR interferes with the normal metabolism of cardiomyocytes, causing cardiomyocyte dysfunction and myocardial fibrosis, whereas autophagy can improve myocardial energy metabolism and protect the normal function of cardiomyocytes. Autophagy alleviates myocardial cell damage caused by IR and T2DM(Ceylan et al., 2018). Therefore, activating autophagy and insulin-signaling pathways, improving insulin sensitivity in diabetic patients, and maintaining an appropriate level of autophagy are important ways to prevent and treat DCM.

**FIGURE 1:**
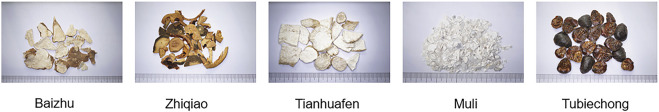
Photographs of *Atractylodes Macrocephala* Koidz. (Baizhu), *Citrus aurantium* L. (Zhiqiao), *Trichosanthes kirilowii* Maxim. (Tianhuafen), *Ostreae Concha* (Muli) and *Eupolyphaga sinensis* Walker (Tubiechong) in medicinal ingredient forms.

Historically, natural products derived from plants and animals have been used for thousands of years in many cultures as a form of traditional medicine. In recent years, the treatment of diabetic complications using traditional Chinese medicine (TCM) has attracted increasing attention (Watal et al., 2014; Bungau and Popa, 2015). In fact, more than 200 traditional medicinal plants and their biologically active compounds have anti-diabetic properties, including reduction of oxidative stress and modulation of autophagy, as confirmed by a large number of screening methods (Yang et al., 2013; Hamza et al., 2020; Abdalla et al., 2021; Nelson et al., 2022). The TCM Yunpi-Huoxue-Sanjie (YP-SJ) formula is based on the famous traditional Chinese prescription Gualou Muli powder and Zhizhu pill, with combination of Tubiechong. Among them, Gualou Muli powder is a famous prescription that was first recorded in the “Synopsis of Prescriptions of the Golden Chamber” written by Zhongjing Zhang of the Han Dynasty (third century A. D.), is composed of Tianhuafen and Muli. Zhizhu pill is a famous formula recorded in “The Theory of Spleen and Stomach” written by Li Dong Yuan of the Jin Dynasty (1249 A.D.), is composed of Baizhu and Zhiqiao. Thus, YP-SJ formula is composed of five natural medicines: *Atractylodes Macrocephala* Koidz. (Chinese name: Baizhu), *Citrus aurantium* L. (Chinese name: Zhiqiao), *Trichosanthes kirilowii* Maxim. (Chinese name: Tianhuafen), *Ostreae Concha* (Chinese name: Muli), and *Eupolyphaga sinensis* Walker (Chinese name: Tubiechong) (Figure 1).

YP-SJ formula has been used to treat diabetic cardiovascular complications for over a decade. According to Chinese medicine theory, *Atractylodes Macrocephala* Koidz. And *Citrus aurantium* L. accelerate the metabolism of sugar and lipids, *Trichosanthes kirilowii* Maxim. And *Ostreae Concha* clear away heat and nourish yin, while the *Eupolyphaga sinensis* Walker accelerates blood flow and both prevents and treats diabetic cardiovascular complications. In our previous randomized controlled clinical trial of YP-SJ formula in combination with other oral hypoglycemic agents to treat DM, we found improvement in IR and vascular inflammation in the combined treatment group relative to the control group (Zhang, X., and Mao, Z.-J., 2018). In animal experiments, YP-SJ formula has been shown to increase serum nitric oxide (NO) levels and reduce serum interleukin-6 (IL-6) levels by activating the phosphoinositide kinase/Akt/endothelial NO synthase pathway (Mao et al., 2020).

TCM has complex components and often exerts clinical effects through multiple pathways and multiple targets. This also increases the difficulty of identifying the active components and clarifying the mechanism of action (Li et al., 2015; Zhang et al., 2015). Pharmacology network is a method that describes complex relationships between biological systems, drugs, and diseases. It has significant applications for studying the mechanisms of multiple components, multiple targets and multiple pathways in TCM. The varied components of YP-SJ formula are complex. Previous studies have determined that YP-SJ formula ameliorates T2DM vascular lesions through an anti-oxidative stress and anti-inflammatory response; however, the specific roles and molecular mechanism remains vague. Therefore, a comprehensive method is applied in this study to illustrate the molecular mechanisms of YP-SJ formula. The active components and mechanism of YP-SJ formula in DCM treatment are predicted using network pharmacology. Afterwards, *in vivo* and *in vitro* experiments are conducted in order to prove the mechanism of its network pharmacological prediction.

## 2 Materials and methods

### 2.1 Chemicals and reagents

The H9c2 cell line was purchased from the cell resource center of Shanghai Institute of Life Sciences, Chinese Academy of Sciences (Shanghai, China). Fetal bovine serum (FBS) (04-001-1ACS, BioInd, Israel) was purchased BioInd, Trypsin (9002-07-7, Gibco, United States), and Dulbecco’s Modified Eagle Medium (DMEM) (C11995500BT,Gibco, United States) were purchased from Gibco. Sodium penicillin and streptomycin sulfate for Meilunboi Co., Ltd. The following primary antibodies were used:β-Actin (ab008, Multi Sciences, China); GAPDH (Mab5465-100, Multi Sciences, China); LC3 II/LC3 I (cst4108, Cell Signaling Technology, United States); Beclin1 (cst4122, Cell Signaling Technology, United States); Atg7 (cst8558, Cell Signaling Technology, United States); FoxO1 (cst2880, Cell Signaling Technology, United States); p-FoxO1 (cst9464, Cell Signaling Technology, United States). Secondary antibodies were purchased from the Multi Sciences.

### 2.2 Preparation of YP-SJ formula

*Atractylodes Macrocephala* Koidz. Granula (Lot No. 19080783), *Citrus aurantium* L. granula (Lot No. 18122333), *Trichosanthes kirilowii* Maxim. Granula (Lot No.19050363), *Ostreae Concha* granula (Lot No.19060963), *Eupolyphaga sinensis* Walker granula (Lot No.19070373), according to“Chinese Pharmacopoeia” (2015 Edition) and internal control quality standards were purchased from Jiangyin Tianjiang Pharmaceutical Co. Ltd. (Jiangyin, China). The granules of *Atractylodes Macrocephala* Koidz., *Citrus aurantium* L., *Trichosanthes kirilowii* Maxim., *Ostreae Concha*, and *Eupolyphaga sinensis* Walker were prepared at a ratio of 5:2:3:10:2 in terms of crude drug content.

### 2.3 Preparation and quality control of YP-SJ formula

#### 2.3.1 Sample preparation

Hesperidin, atractylodin, hypoxanthine, cucurbitacins B, and taurine (0.2 g each) were separately added to different 5-ml centrifuge tubes with chromatographic methanol (34860, Sigma-Aldrich, United States) and sonicated for 15 min. Then 0.2 ml solution was filtered using a 0.22-µm filter, and the filtrate was collected and centrifuged at 18000 r/min for 5 min. Finally, 20 µl of the supernatants for each compound was used for high-performance liquid chromatography (HPLC) (20AT, Shimadzu, Japan). The reference standards of Hesperidin, atractylodin,hypoxanthine, cucurbitacins B, and taurine were purchased from Shanghai Yuanye Bio-Technology Co., Ltd (Shanghai, China). Its purity is greater than or equal to 98%

### 2.3.2 Chromatographic conditions

The chromatographic conditions were as follows.

Hesperidin: mobile phase, methanol-0.1% phosphoric acid solution (36:64, v/v); flow rate,1.0 ml/min; column temperature, 25°C; ultraviolet detection wavelength, 286 nm; and injection volume,20 μl.

Atractylodin: mobile phase, acetonitrile-water (70:30, v/v); flow rate,1.0 ml/min; column temperature,35°C;ultraviolet detection wavelength, 340 nm; and injection volume,20 μl.

Hypoxanthine: mobile phase, methanol-0.1% phosphoric acid solution (2:98, v/v); flow rate, 0.5 ml/min; column temperature, 25°C; ultraviolet detection wavelength, 254 nm; and injection volume, 20 μl.

Cucurbitacins B: mobile phase, methanol-0.1% phosphoric acid solution (53:47, v/v); flow rate, 1.0 ml/min; column temperature, 25°C;ultraviolet detection wavelength, 254 nm; and injection volume, 20 μl.

Taurine: mobile phase, methanol-0.05 M sodium acetate solution (40:60, v/v); flow rate, 0.8 ml/min; column temperature, 25°C; ultraviolet detection wavelength, 330 nm; and injection volume, 20 μL.

A mixture was made of sodium borate buffer (0.6M; 3.72 g boric acid (Wuxi Zhanwang Chemical Reagent Co., Ltd., Wuxi, China) and 2.1 g sodium hydroxide(Hangzhou Xiaoshan Chemical Reagent Factory, Hangzhou, China)), derivatization reagent (0.1 g o-phthalaldehyde), 10 ml chromatographic methanol, and 0.1 ml mercaptoethanol(Biotech, Germany).

### 2.4 Network pharmacology

#### 2.4.1 Component collection

The Traditional Chinese Medicine Systems Pharmacology Database and Analysis Platform (TCMSP, https://tcmspw.com/tcmsp.php) is an efficient database for systems pharmacology research regarding TCM. The chemical composition information of YP-SJ formula was supplemented by TCMSP. Oral bioavailability (OB) refers to the percentage of oral drugs reaching systemic circulation, which is one of the pharmacokinetics important indicators for drug screening. The OB threshold is set to be ≥30%. Drug likeness (DL) represents the degree of “drug similarity” of a target compound and is used to remove chemically inappropriate compounds. TCMSP uses the Tanimoto similarity method to calculate the DL index by comparing the target compound with 6651 molecules in the DrugBank database. We set the DL threshold to≥ 0.18.

The Integrative Pharmacology-based Research Platform of Traditional Chinese Medicine (TCMIP, http://www.tcmip.cn/) v2.0 is based on the database resources of the Encyclopedia of Traditional Chinese Medicine (ETCM, http://www.tcmip.cn/ETCM/index.php/Home/Index/), which can effectively reveal the material basis of TCM and its molecular mechanism in the data mining platform. The Latin names of the five TCM in YP-SJ formula were entered into the database, all chemical components were retrieved, and the chemical components were screened by a quantitative estimate of drug-likeness (QED). The ADMET collection model calculates the QED score and retains components with a QED score of medium and high (QED ≥0.49). Supplemented with SymMap (https://www.symmap.org/), HERB (http://herb.ac.cn/) databases.

#### 2.4.2 Target prediction

The names of compounds collected above were normalized in Pubchem and the SMILES numbers were collected and inputted into the Swiss Target Prediction database (http:∥swisstargetprediction.ch/) for target prediction, and the reliability card value was greater than 0.25. At the same time, the Related targets function of the TCMSP platform was used to collect potential targets. To improve the credibility of the predicted targets, the total targets collected in the two databases were intersected, the compounds without corresponding targets were deleted, and the network relationship between TCM-compound-targets was input into Cytoscape, and the network diagram was drawn.

#### 2.4.3 Construction of protein-protein interaction network

The STRING database (https://string-db.org/) is a database for searching known protein interaction relationships, which provide an effective way to construct protein-protein interaction (PPI) networks. In order to illustrate the gene expression of the intersection target genes, the potential target genes are imported into the String database, the species is set to human (*Homo sapiens*), the confidence threshold is set to 0.4, and free proteins are removed to obtain the PPI network of the potential target genes, which will be obtained. The PPI network data are input into Cytoscape for visualization processing, and the complex network relationship is analyzed through the Analyze Network module to obtain the degree of connectivity. Through this parameter, we can digitize complex network relationships to obtain core proteins.

#### 2.4.4 Gene Ontology and Kyoto Encyclopedia of Genes and Genomes enrichment analysis

Enter the Bioconductor database for Gene Ontology (GO) and Kyoto Encyclopedia of Genes and Genomes (KEGG) enrichment analysis, calculate the *p*-value based on the cumulative hypergeometric distribution, and use the BanjaminiHochberg program to correct the *p*-value, taking *p* < 0.05 to screen out the most representative enrichment results(Xie et al., 2021). Deletion of signaling pathways less relevant to diabetic cardiomyopathies, such as cancer-related signaling pathways, from KEGG pathway analysis.

### 2.5 *In Vivo* experiment

#### 2.5.1 Animal

Twenty-four healthy male specific-pathogen-free (SPF) Wistar rats weighing 300–350 g was purchased from the Shanghai SLAC Experimental Animal Co., Ltd. The rats were housed in a SPF conditions-controlled temperature (25 ± 1°C) and humidity (50 ± 5%) environment with a 12-h light/dark cycle and allowed free access to sterilized food and water. All animal procedures followed the Chinese Animal Welfare Law (certificate number: 20170005026461).

#### 2.5.2 DCM rat model

After acclimatization for 1 week, the rats were randomly divided into two groups: the control group (n = 8) and the model group (n = 16). The control group was given a normal diet, and the model group was given WD12492 high-fat diet. From the third week, the rats in the model group were given an intraperitoneal injection of streptozotocin (STZ) 30 mg/kg at one time, and the rats in the control group were intraperitoneally injected with the same volume of citrate buffer (Mansor et al., 2013). In the sixth week, the blood of fasting rats was collected, fasting plasma glucose (FPG) and fasting plasma insulin (FINS) were detected, and homeostatic model assessment for IR (HOMA-IR) was calculated. FPG >11.1 mmol/L and IR indicated successful modeling (Le Page et al., 2015).

The 16 rats in the model group were randomly divided into a model group and the treated group, with eight rats in each group. The model group was orally administered with 10 mg/kg of normal saline once a day. The treated group was orally administered with 10 ml/kg (12g/10 ml) YP-SJ formula (crude drug amount 12 g/kg). Dosing was continued for 10 weeks.

#### 2.5.3 Dose-response relationship of YP-SJ formula on DCM rats

In order to study the dose-effect relationship, we divided 30 male Wistar rats into a control group (n = 5) and a model group (n = 25), and subsequently divided the successfully modeled rats into a control group, YP-SJ formula Dosage group (6 g/kg, 12 g/kg, 24 g/kg). Next, we determined the optimal dosage by measuring myocardial malonaldehyde (MDA), catalase (CAT), free fatty acid (FFA), triglyceride (TG), and other indicators and tissue structure change.

#### 2.5.4 Assessment of cardiac function

Two-dimensional echocardiography was used to determine cardiac function and heart dimensions using a high-resolution small-animal imaging system (Vevo1100; Visual Sonics, Toronto, Canada) while the rats were anesthetized with 1.5–2% isoflurane. The system was equipped with a high-frequency ultrasound probe (MS250). The measurements of left ventricular end-systolic diameter (LVESD) and left ventricular end-diastolic diameter (LVEDD) were based on the analysis of at least three separate cardiac cycles. Fractional shortening (FS) was calculated according to the following formula: FS (%) = [(LVEDD−LVESD)/LVEDD] ×100. Ejection fraction (EF%) was calculated by: EF (%) = ([LVEDD − LVESD]/LVESD) ×100. Mitral valve peak flow velocity was measured in the early diastole (E) and late diastole (A), and the E/A ratio was calculated.

#### 2.5.5 Collection of heart samples

After the cardiac function test, the rats were weighed and anesthetized by intraperitoneal injection of sodium pentobarbital (40 mg/kg), the abdominal cavity was opened, and the rat heart was taken out. Death was confirmed by the loss of pinch reflexes and rigor mortis after exsanguination following heart removal in rats. During this process, the mouse did not struggle and be very quiet.

#### 2.5.6 Western blotting

Western blot was used to detect the expression levels of LC3, Beclin1, Atg7, Foxo1, and p-FoxO1 proteins in the myocardial tissue of rats in each group. Myocardial tissue was cut into pieces and homogenized in RIPA buffer. The tissue homogenate was incubated on ice for 20 min and centrifuged at 11000 rpm for 20 min at 4°C. Transfer the supernatant to a new 1.5 ml centrifuge tube and store at -80°C live, and the protein content in the tissue extract was measured with a BCA protein assay kit according to the manufacturer’s instructions. An aliquot of the protein solution was mixed with loading buffer and heated at 100°C for 10 min. The protein solution was separated by sodium dodecyl sulfate-polyacrylamide gel electrophoresis (12%) and transferred to a polyvinylidene difluoride membrane.

#### 2.5.7 Histological analysis

Myocardial tissue was fixed with 4% paraformaldehyde, embedded in paraffin, and cut into 6-μm sections. Myocardial inflammation and necrosis in DCM rats were observed with hematoxylin and eosin (H&E), and Masson trichomes were stained in DCM rats of myocardial fibrosis, and lipid distribution in cardiac tissue examined by Oil Red O staining.

Rat myocardial tissue was cut into 1-mm^3^ tissue blocks, fixed in 2.5% glutaraldehyde at 4°C for 2 h, and washed twice with phosphate-buffered saline (PBS) at 4°C for 15 min each. Then the tissues were stained and fixed in 1% osmium acid at 4°C for 1 h, rinsed again with PBS, and stained with 2% uranyl acetate solution for 30 min. After gradient ethanol dehydration, propylene oxide replacement, embedding with pure embedding agent, polymerization in an oven, and staining with 4% uranium acetate, transmission electron microscopy (TEM) (TECNA-10; Netherlands Philips Co., Amsterdam, Netherlands) was performed.

### 2.6 *In vitro* experiment

#### 2.6.1 Preparation of YPSJ-containing serum

Thirty rats were randomly divided into two groups. The blank control group was given normal saline, and the YP-SJ group was given YP-SJ (12 g/kg/day) for seven consecutive days. Rats were anesthetized with sodium pentobarbital (Sigma, USA) intraperitoneally, and abdominal aortic blood was collected from rats under sterile conditions 1 h after YP-SJ or saline administration, followed by serum extraction. The centrifuged serum was inactivated at 56°C for 30 min. The resulting serum was filtered through a microporous membrane to remove bacterial contamination before use and stored at -80°C for later use (Yu et al., 2021).

#### 2.6.2 Cell culture and treatment

H9c2 cells were cultured in DMEM medium containing 10% FBS and 1× penicillin (69-57-8, meilunbio, Dalian, China)-streptomycin (3810-74-0, meilunbio, Dalian,China) in a 37°C, 5% CO_2_, saturated humidity incubator. They were divided into six groups, namely control group, high glucose (HG) group, high glucose + control serum (HG + CS) group, high glucose + dosed serum (HG + DS) group, high glucose + NC-siRNA (NC-siRNA) group, high glucose + siRNA-FoxO1 (siRNA-FoxO1) group.

#### 2.6.3 Flow cytometry assay

Cells were digested with trypsin (no ethylenediaminetetraacetic acid) into a single-cell suspension and resuspended in binding buffer. The cells were then stored at room temperature. Using an Annexin V-FITC/PI apoptosis kit (AP101C, Multi Sciences, Hangzhou, China), the cells were stained for 15 min without light, and the rate of apoptosis was detected using BD accuri C6 flow cytometry (ACCURI C6, BD Biosciences, United States).

#### 2.6.4 Immunofluorescence analysis

The normal cultured H9c2 cells in the log phase were taken, digested with 0.25% Trypsin, centrifuged at 1,000 rpm for 5 min, counted under a counting plate, spread on a 24-well plate, and 8 × 10 ^4^ cells were added to each well, for a total of six wells. Incubate in an incubator. Cells were grown on a coverslip. The cells were then fixed in 4% paraformaldehyde for 15 min, followed by incubation with permeabilization solution for 30 min at room temperature. The coverslip was washed with PBS three times and incubated with a blocking solution for 1 h at room temperature. Primary antibody in blocking solution was added, cells were incubated overnight at 4°C, washed with blocking solution, and then incubated secondary antibody for 1.5 h at room temperature. The coverslip was washed and mounted with 10 ng/ml DAPI (D9542, sigma, United States) stain and visualized using a fluorescence microscope (IX73, OLYMPUS, Japan).

#### 2.6.5 Observation of ultrastructure and autophagosomes of cardiomyocytes

Cells were digested, washed with PBS after centrifugation, fixed with 2.5% glutaraldehyde for 4 h, and washed with PBS. Then was subsequently fixed in 1% osmium acid for 30 min–1 h and dehydrated in ethanol and acetone. Finally, it was embedded in epoxy resin and fixed and stained with uranyl acetate solution. The TEM was used to observe the changes in autophagy morphology.

#### 2.6.6 Western blotting

Cells were grown on 6-well culture plates and were treated with the specific compounds for the indicated time. The cells were then lysed with lysis buffer (P0013, Beyotime, China), and protein concentrations were determined by the BCA assay (P0012S, Beyotime, China). The same amount of lysate proteins were separated by electrophoresis on SDS-polyacrylamide gels and electroblotted onto polyvinylidene difluoride membrane (IPVH00010, Millipore, United States). The membranes were probed with specific primary antibodies and appropriate secondary antibodies. The immunoreactive bands were visualized using an ECL detection kit (P0018FS, Beyotime, China).

### 2.7 Statistical analysis

Data were expressed as means ± SEM. Student’s t-test or one-way analysis of variance (ANOVA) followed by the Bonferroni post hoc test was used for two or multiple comparisons. Statistical significance was accepted at *p* < 0.05.

## 3 Results

### 3.1 Optimal dosage of YP-SJ formula

#### 3.1.1 YP-SJ formula attenuates cardiac hypertrophy and fibrosis in DCM rats

**FIGURE 2:**
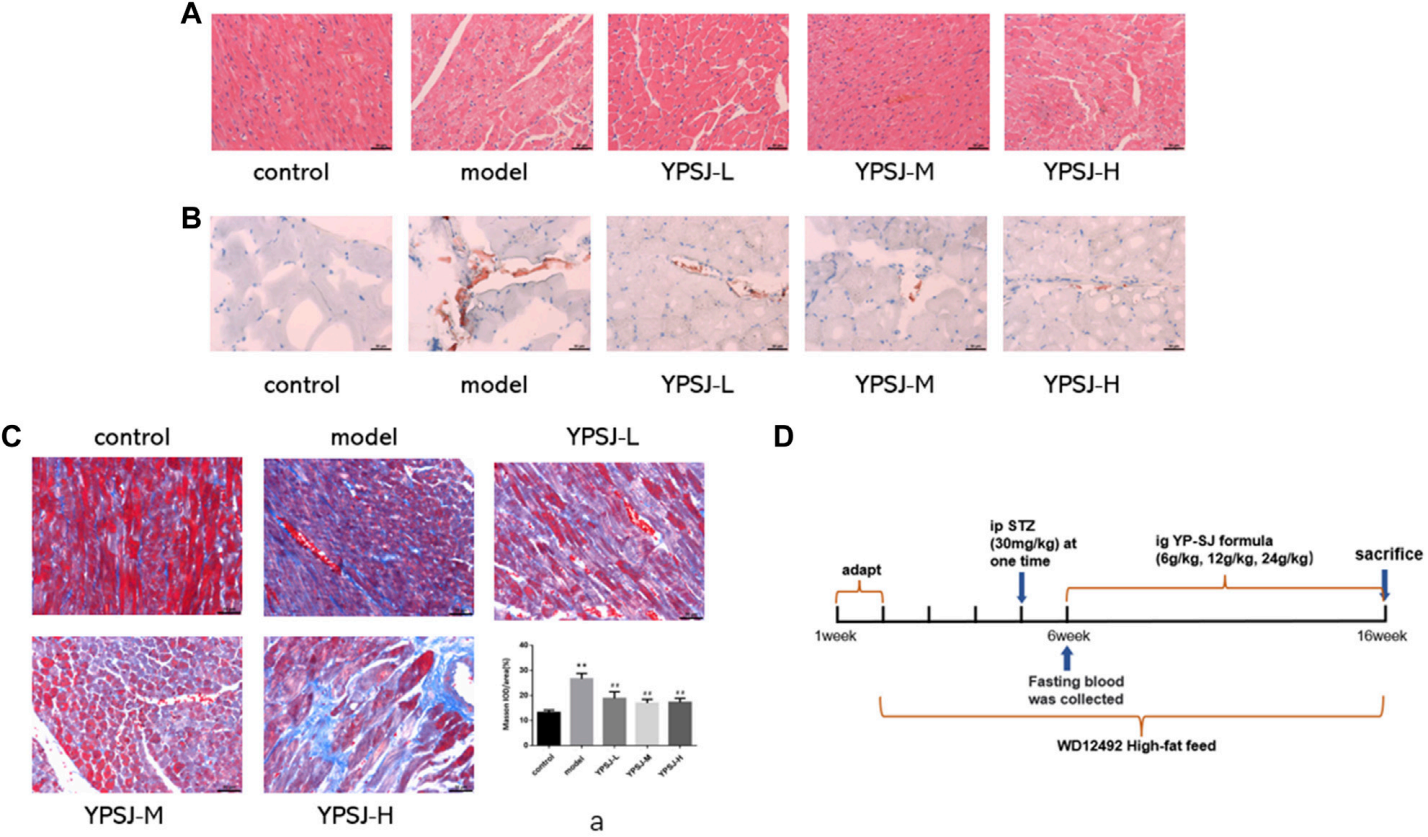
The effect of Yunpi-Huoxue-Sanjie (YP-SJ) formula on the rat myocardium. Myocardial specimens (n = 5) were collected and stained with H&E (×200) **(A)**; The effect of YP-SJ formula on myocardial lipid accumulation in rats in each group. Myocardial specimens (n = 5) were collected and stained with Oil red O staining (×200) **(B)**; The effect of YP-SJ formula on myocardial fibrosis in rats of each group. Myocardial specimens (n = 5) were collected and stained with Masson staining (×200) **(C)**; Animal experiment time chart **(D)**; **p < 0.01 vs. control group; ##*p* < 0.05 vs. model group (a).

In order to determine the role of YP-SJ formula in DCM, we used a well-established STZ-induced type 2 diabetes rat models and started to inject STZ intraperitoneally in the third week of modeling. After successful modeling, the YP-SJ formula group was tested 10 weeks of dosing (Figure 2D). Myocardial cells in the YP-SJ formula group were slightly edematous, vacuoles were seen in a few cells, and inflammatory cell infiltration was occasionally seen. Compared with the model group, myocardial pathological damage was significantly reduced (Figure 2A). In addition, there was a small amount of fat deposition between myocardial fibers in each group of YP-SJ formula, which was significantly less than that in the model group (Figure 2B). Meanwhile, control group, the interstitial collagen fibers were less distributed and evenly distributed. The collagen fibers in the myocardial tissue of the model group increased significantly, and the distribution was disordered and uneven. Compared with the model group, myocardial collagen fibers in the 6 g/kg, 12 g/kg, 24 g/kg groups (YPSJ-L, YPSJ-M and YPSJ-H) of YP-SJ formula were significantly reduced (Figure 2C). In conclusion, YP-SJ formula is protective against myocardial injury in DCM rats.

#### 3.1.2 YP-SJ formula improves myocardial MDA, CAT, FAA, and TG levels in DCM rats

**FIGURE 3:**
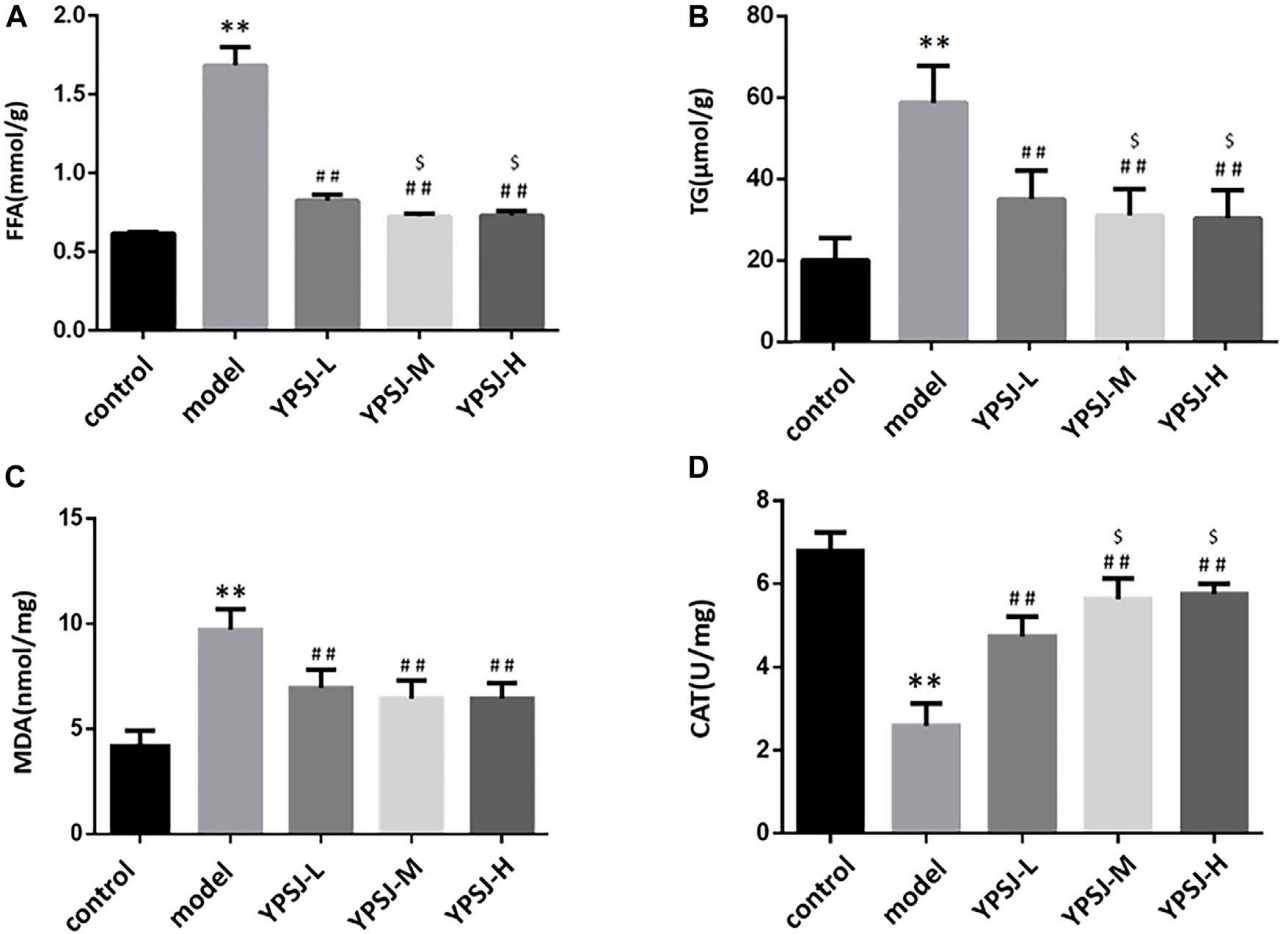
Comparison of myocardial free fatty acid (FFA) and triglyceride (TG) levels in each group of rats **(A,B)**; Comparison of myocardial malonaldehyde (MDA) and catalase (CAT) levels in each group of rats **(C,D)**; **p < 0.01 vs. control group; ##*p* < 0.01 vs. model group; $*p* < 0.05 vs. YPSJ-L group.

In DCM, myocardial glucose oxidative utilization decreases, while fatty acid oxidation rate increases, and lipid droplets such as triglycerides and FFA accumulate in cardiomyocytes, resulting in unbalanced volume distribution in the cytoplasm and affecting myocardial diastolic and systolic function. Fatty acids are an important source of cellular energy under starvation conditions, but excessive FFA accumulation in the cytoplasm can cause lipotoxic damage to cells (Li et al., 2017). Therefore, we can detect whether YP-SJ influences DCM rats through the levels of FFA and TG. Compared with the control group, the levels of myocardial FFA and TG in the model group were significantly increased (*p* < 0.01) (Figures 3A,B); compared with the model group, the levels of FFA and TG in the low-dose, middle- and high-dose groups of YP-SJ formula were significantly decreased (*p* < 0.01). Compared with the low dose, the FFA and TG levels in the middle and high dose groups of YP-SJ formula were significantly decreased (*p* < 0.05); there was no statistical difference between the middle dose and the high dose (*p* > 0.05).

Under a high glucose environment, abundant reactive oxygen species produced by mitochondria induce oxidative damage as an important driver of diabetes progression of DCM(Pickering et al., 2018). MDA is one of the main products of lipid peroxidation, and its expression level can directly reflect the rate and degree of lipid peroxidation (Schettler et al., 1999); CAT can protect cells from the poisoning of hydrogen peroxide and is one of the key enzymes in the biological defense system. The content level can reflect the degree of damage to cells caused by oxidative stress. Compared with the control group, the myocardial MDA level in the model group was significantly increased (*p* < 0.01), and the CAT level was significantly decreased (*p* < 0.01). Compared with the model group, MDA levels were significantly decrease and CAT levels were significantly increase in the low, middle, and high doses of YP-SJ formula (*p* < 0.01); compared with the low dose group of YP-SJ formula, there was no significant difference in MDA between the middle and high dose groups of YP-SJ formula (*p* > 0.05), CAT levels were significantly different (*p* < 0.05) (Figures 3C,D).

According to the above experimental results, it is determined that the optimal effective dose of the drug is 12 g/kg.

### 3.2 Potential pharmacological mechanisms of YP-SJ formula

#### 3.2.1 Drug-ingredient-target gene network

**FIGURE 4:**
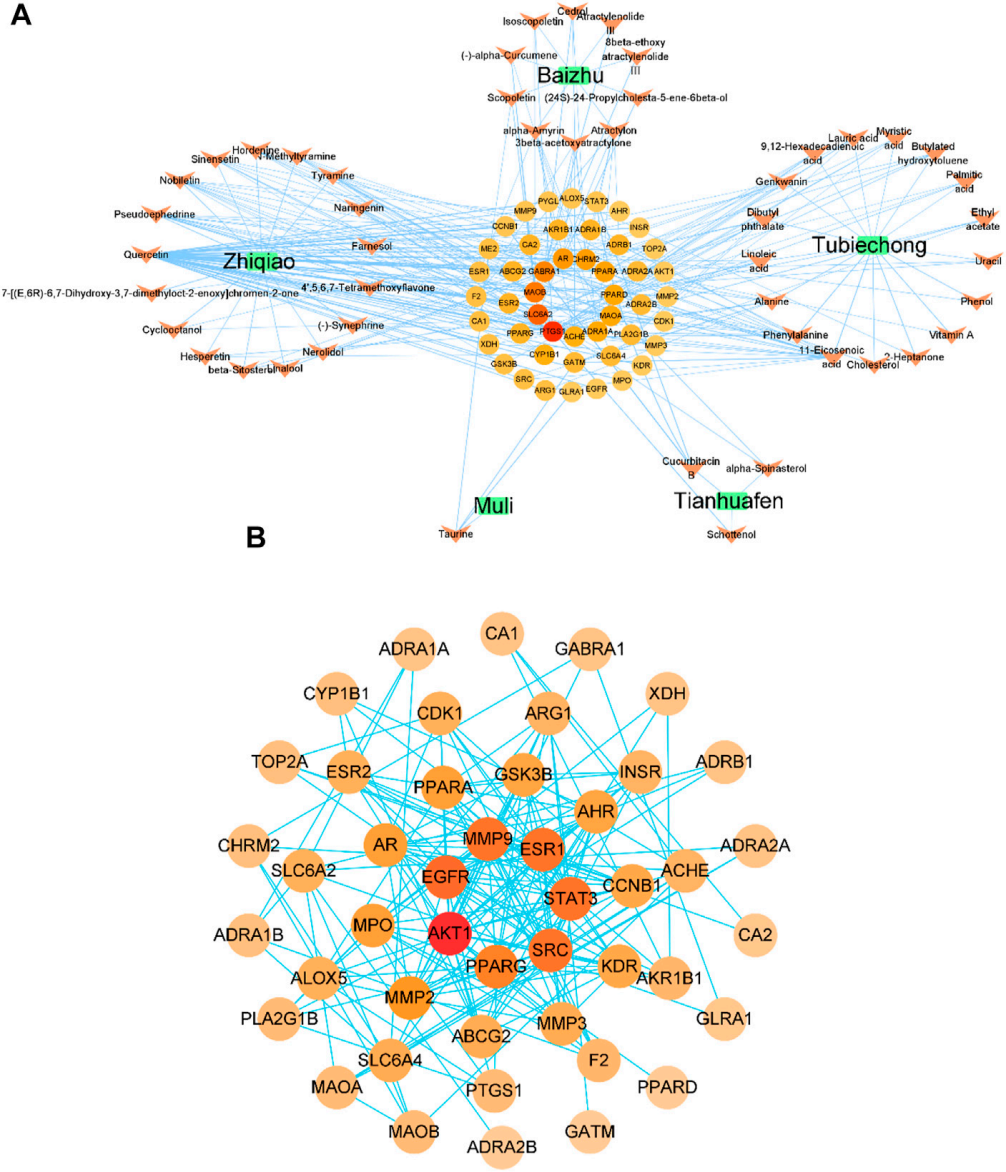
Drug-ingredient-target gene network diagram **(A)**; protein-protein interaction (PPI) network and core protein network, the color and size of the nodes depend on the connectivity value **(B)**.

By combining TCMSP and TCMIP databases of TCM, removing duplicates and standardizing names, and screening based on their pharmacokinetics properties (with OB, DL, BBB as the measurement indicators), 10 active ingredients of Atractylodes Rhizoma and 17 of Citrus aurantium were obtained. The active ingredients, terrestrial softshell worm, trichosanthin, and oyster got 17, 3, and one respectively. We obtained 46 potential target genes by combining the Swiss Target Prediction tool and the TCMSP database, and then the data processing of our Excel tool and the visual analysis of Cytoscape finally obtained the drug-component-target gene network diagram (Figure 4A).

Green squares represent drugs, orange arrows represent active ingredients, and circles represent potential target genes.

#### 3.2.2 Protein-protein interaction network

The above targets were input into the String platform for PPI network analysis, with nodes representing target genes and edges representing the linkage between target genes. 46 nodes of YP-SJ formula target genes (no free targets), 193 edges, an average node degree of 8.39 and an average local clustering coefficient of 0.595 were obtained. Network data analysis revealed a strong association between the target genes of YP-SJ formula for DCM treatment. To screen out the core target genes in the PPI network, the network data were input into Cytoscape for visualization (Figure 4B), and the central properties of the target genes were evaluated based on topological analysis, and the results showed that AKT1, EGFR, SRC, MMP9, ESR1, STAT3, PPARG and other proteins are the core proteins in the PPI network and play an important role in regulating the network role in the regulatory network.

#### 3.2.3 GO enrichment analysis results

**FIGURE 5:**
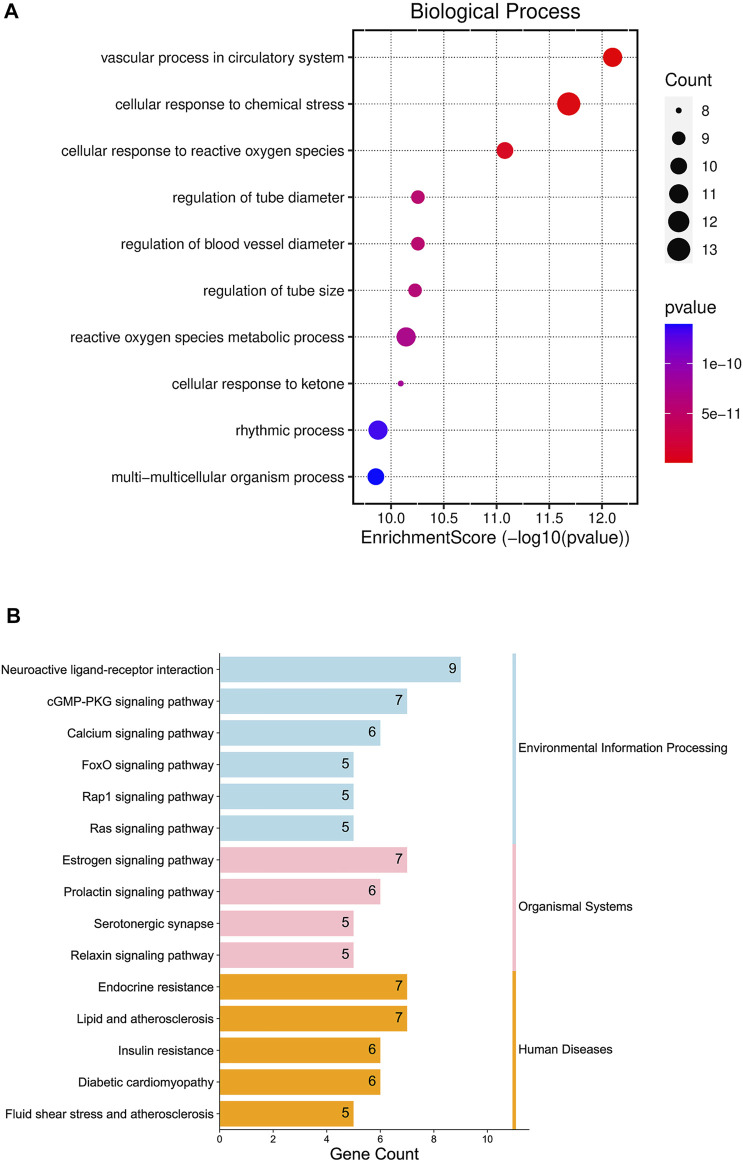
Gene Ontology (GO) enrichment analysis diagram **(A)**; Kyoto Encyclopedia of Genes and Genomes (KEGG) Enrichment Pathway Bar Chart **(B)**.

Enter the Bioconductor database for GO enrichment analysis (take *p* < 0.05), and obtain a total of 1,364 representative functional clusters, which are sorted according to the number of target genes involved, and the first 10 enrichment results are retained for analysis. The top ten items of go enrichment analysis results are multi-multicellular organism process, rhythmic process, cellular response to ketone, reactive oxygen species metabolic process, regulation of tube size, regulation of blood vessel diameter, regulation of tube diameter, cellular response to reactive oxygen species, cellular response to chemical stress and vascular process in the circulatory system (Figure 5A).

#### 3.2.4 KEGG enrichment analysis results

**FIGURE 6:**
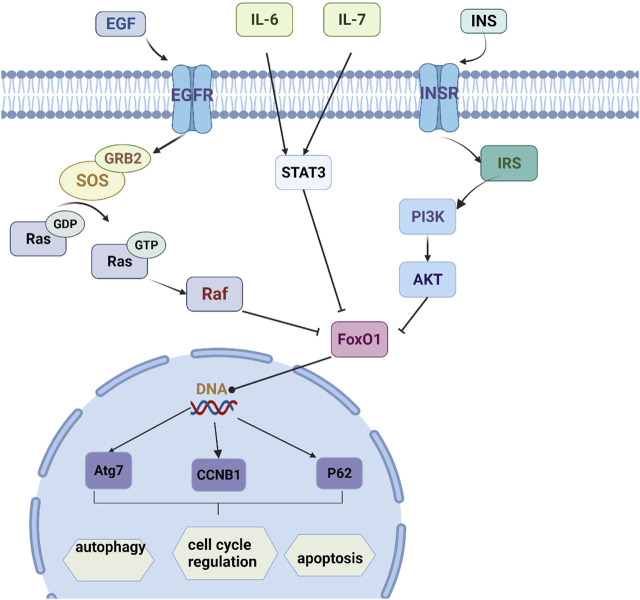
Mechanism pathway map.

The Bioconductor database was used for KEGG enrichment analysis (*p* < 0.05), and a total of 93 representative functional clusters were obtained. Removal of signaling pathways less associated with insomnia. For example, Acid kinase inhibitor resistance (EGFR tyrosine kinase inhibitor resistance, hsa01521), breast cancer (Breast cancer, hsa05224), non-alcoholic fatty liver disease (Non-alcoholic fatty liver disease, hsa04932), etc., and according to the number of target genes involved Sort and retain the results involving ≥5 target genes, a total of 15 (Figure 5B). By analyzing the pathway results and literature, several pathways with high analytical value were selected for sorting and drawn into a pathway map of YP-SJ formula treating DCM through the target of FoxO1 (Figure 6).

### 3.3 HPLC analysis of the YP-SJ formula

**FIGURE 7:**
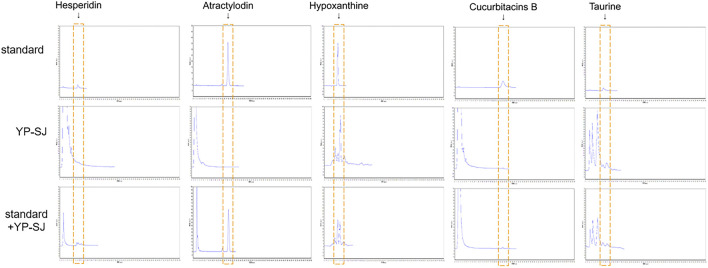
HPLC estimation of YP-SJ formula. The presence of hesperidin, atractylodin, hypoxanthine, cucurbitacins B and taurine were adopted as quality controls for YP-SJ formula. The abovementioned ingredients were detected in standard, YP-SJ and standard + YP-SJ samples.

The retention time of hesperidin was about 8.898 min. The content of hesperidin in the granules was 0.054 ± 0.0029% (n = 3); After extraction of methanol-dissolved granules, the content of hesperidin was 0.066 ± 0.0078% (n = 3). Atractylodes are highly soluble in fat and insoluble in water; thus, no atractylodin was detected in the aqueous extract of the Chinese herbal compound. The retention time of hypoxanthine was about 6.985 min. The content of hypoxanthine in the granules was 0.019 ± 0.00058% (n = 3). The retention time of Cucurbitacin B was about 23.885 min. The content of Cucurbitacin B in the granules was 0.0022 ± 0.00045% (n = 3). After extraction of methanol-dissolved granules, the content of hesperidin was 0.0044 ± 0.00025% (n = 3). The retention time of taurine derivatives was about 8.368 min. The content of taurine in the granules was 0.15 ± 0.0058% (n = 3), as shown in the chromatogram Figure 7.

### 3.4 Results of in vivo experiments

#### 3.4.1 YP-SJ formula attenuates IR in DCM rats

**FIGURE 8:**
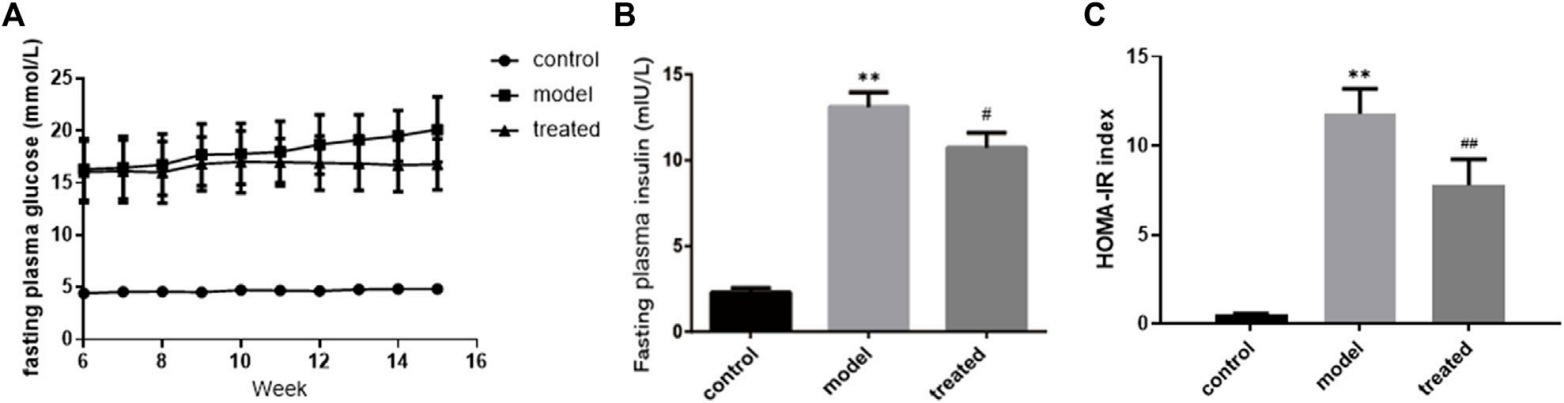
The effect of YP-SJ formula on **(A)** fasting plasma glucose, **(B)** fasting plasma insulin and **(C)** HOMA-IR index. Data are shown by mean ± SEM (n = 8). ^**^ *p* < 0.01 vs. control group; ^##^ *p* < 0.01 and ^#^ *p* < 0.05 vs. model group.

Compared with the control group, the FPG of rats in the model group increased by 4.2-fold. After YP-SJ formula treatment, compared with the model group, levels were reduced by 16.6% (Figure 8A). Before the end of the experiment, the HOMA-IR index was higher in the model group than in the control group. Compared with the model group, after YP-SJ formula treatment, FINS and HOMA-IR indexes were reduced by 18.1% and 33.9%, respectively (Figures 8B,C).

#### 3.4.2 YP-SJ formula improves cardiac function in DCM rats

**FIGURE 9:**
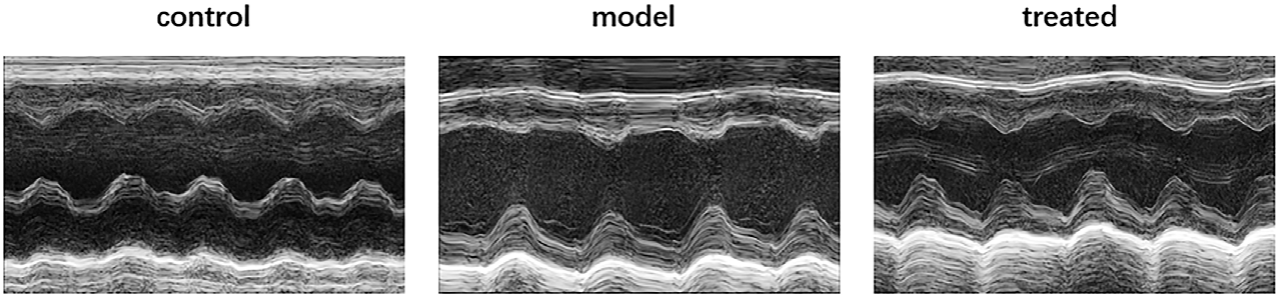
YP-SJ formula improves cardiac function in Diabetic cardiomyopathy (DCM) rats.

**FIGURE 10:**
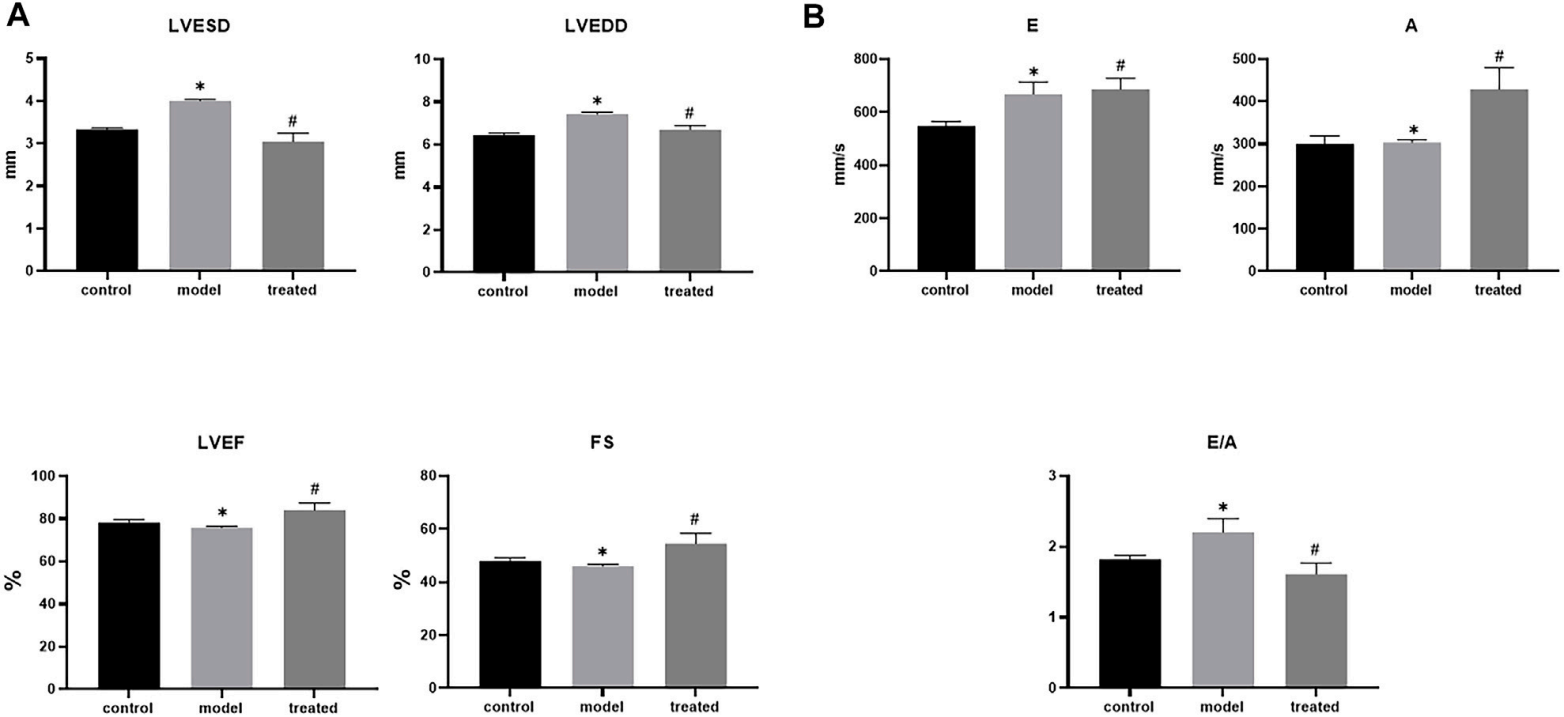
Each group left ventricular end-systolic diameter (LVESD), left ventricular end-diastolic diameter (LVEDD), Fractional shortening (FS), left ventricular ejection fraction (LVEF). **(A)**; early diastole (E), late diastole (A) and E/A of rats in each group **(B)**, Data are shown by mean ± SEM (n = 8). ^*^ *p* < 0.05 vs. control group; ^#^ *p* < 0.05 vs. model group.

Compared with the control group, the model group had impaired cardiac function (Figure 9), mainly manifested as increased LVESD, LVEDD, and LVEF, FS decreased (Figure 10). But these indicators were significantly improved in the treated group. From the above data, it can be found that YP-SJ gavage can rescue cardiac dysfunction.

#### 3.4.3 Transmission electron microscopy

**FIGURE 11:**
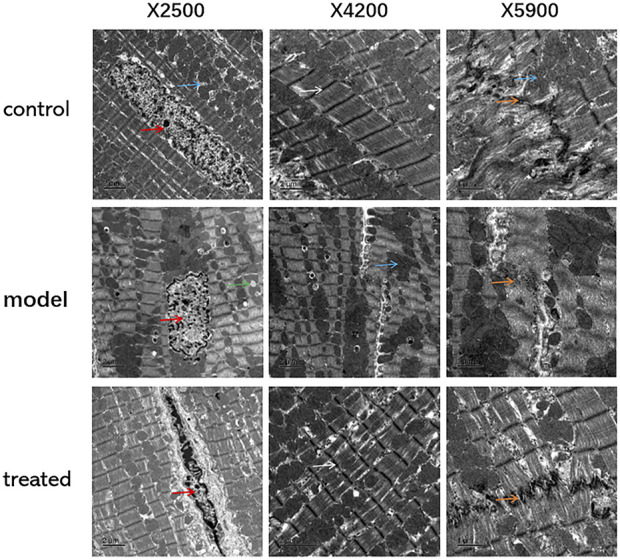
The effects of YP-SJ formula on myocardial autophagy in DCM rats (Transmission electron microscopy, TEM), (Its magnification is×2500, ×4200, and ×5900 respectively).

The results of TEM showed that, compared with the control group, the myocardial tissue of the model group had swollen mitochondria, increased volume, cristae moved to the periphery, focal vacuoles appeared in part of the mitochondrial matrix, matrix material was lost, and part of the mitochondrial membrane was visible. Rupture; autophagosomes decrease (Figure 11).

Cardiomyocytes are shown by red arrows, mitochondria are shown by blue arrows, intercalated discs are shown by orange arrows, lipid droplets are shown by green arrows, and Z lines are shown by white arrows.

#### 3.4.4 Effects of YP-SJ formula on protein expression in the heart of DCM rats

**FIGURE 12:**
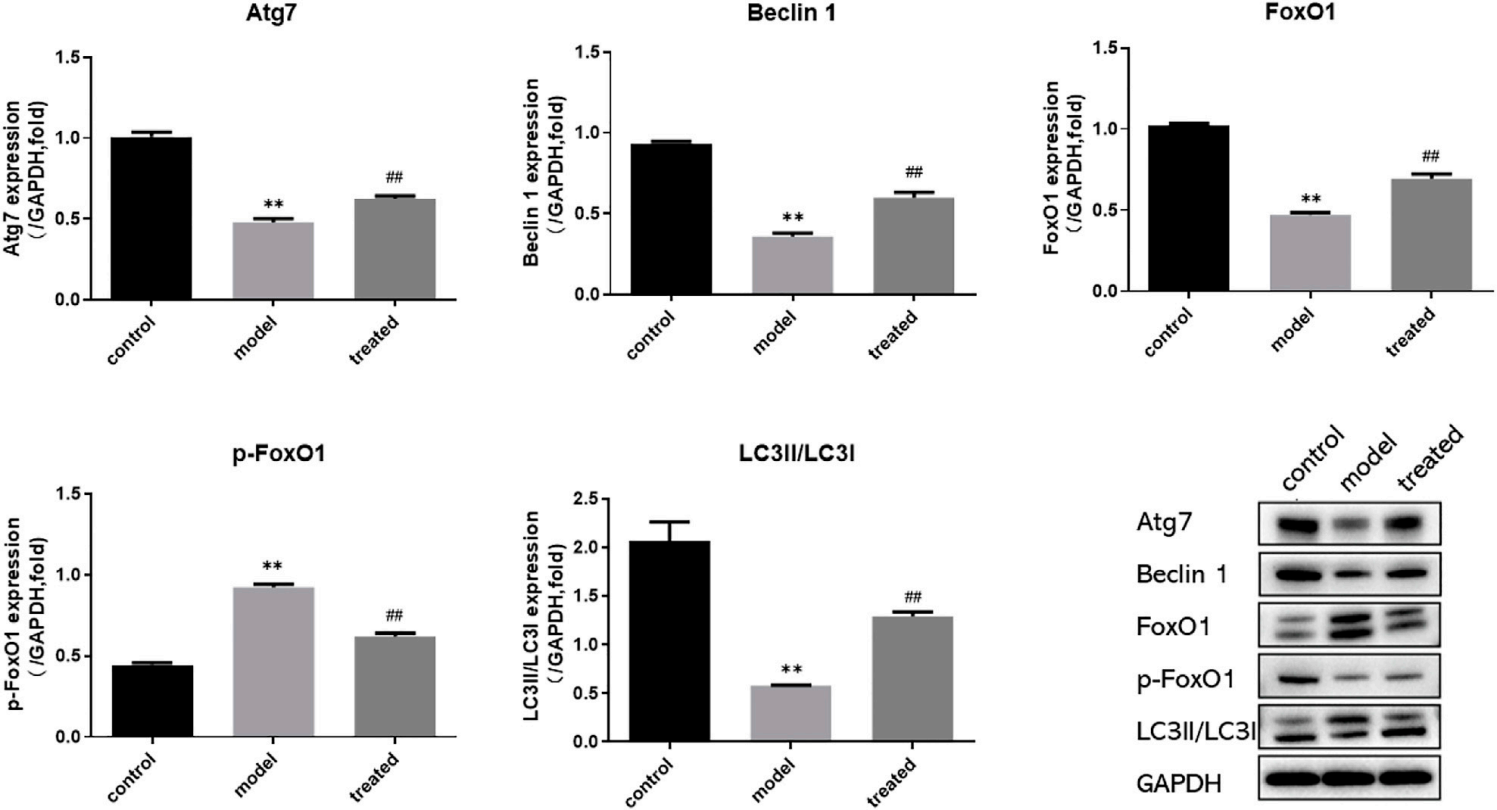
Western blot analysis of Atg7, FoxO1, p-FoxO1, Beclin1, LC3 and GAPDH protein expression in the heart. ^**^ *p* < 0.01 vs. control group; ^##^ *p* < 0.01 vs. model group.

Compared with the control group, the protein expression of Atg7, FoxO1, Beclin1, LC3 II/LC3 I in the model group were significantly decreased (*p* < 0.01), and the protein expression of p-FoxO1 was significantly increased (*p* < 0.01); Compared with the model group, the protein expression of Atg7, FoxO1, Beclin1, LC3 II/LC3 I were significantly increased (*p* < 0.01), and p-FoxO1 protein levels were significantly decreased in the treated group (*p* < 0.01). (Figure 12).

In conclusion, YP-SJ formula administration retained cardiac function in DCM rats and ameliorated myocardial injury by upregulated FoxO1 expression after disease onset.

### 3.5 Results of in vitro experiments

#### 3.5.1 YP-SJ formula inhibits H9c2 cell apoptosis

**FIGURE 13:**
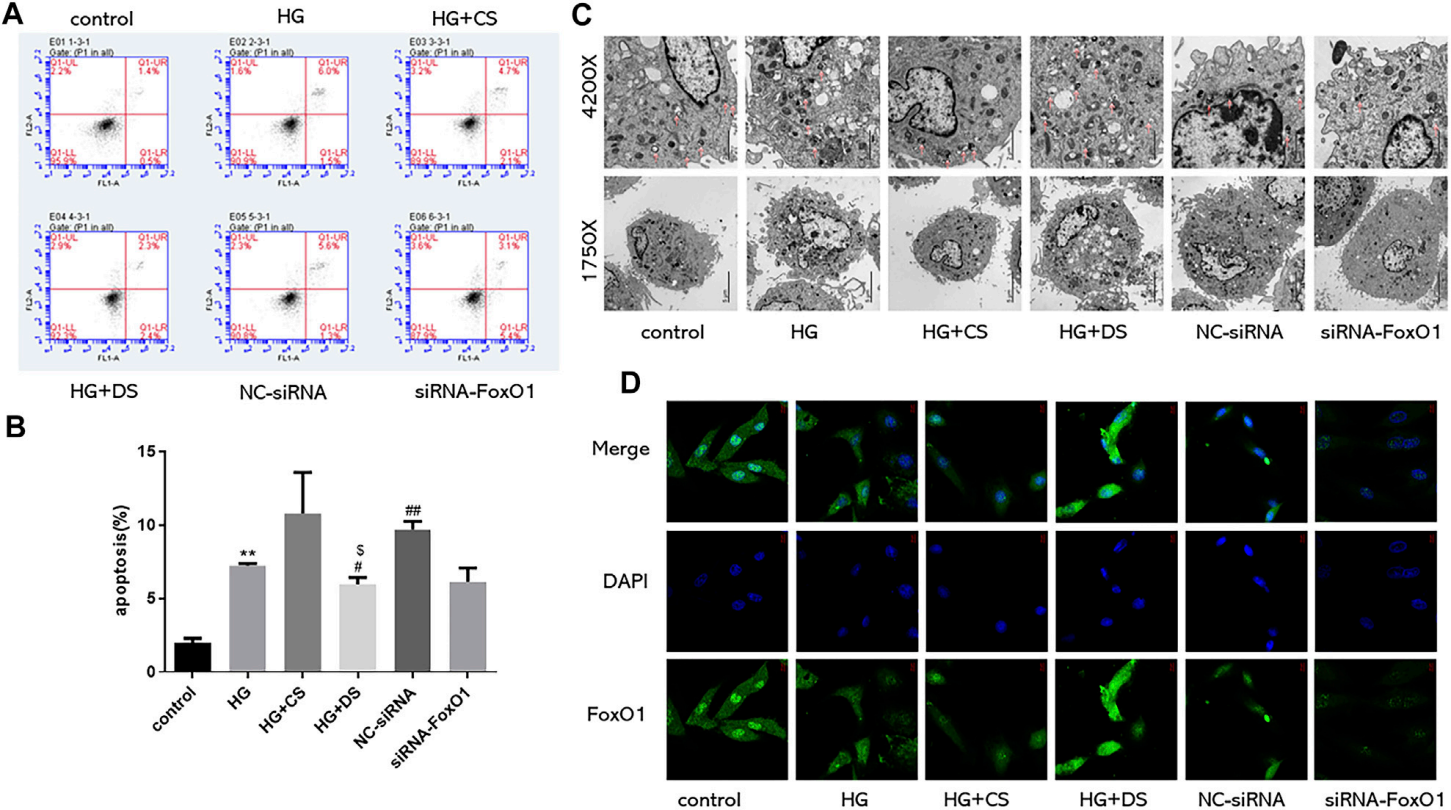
Flow cytometry was used to detect the proportion of apoptotic cells (n = 3) **(A,B)**; Observation of autophagosomes in H9c2 cells under electron microscope (Magnification of ×1750 and 4200× respectively) **(C)**; Immunofluorescence of FoxO1 expression in H9c2 cells under fluorescence microscope (630×) **(D)**. **p < 0.01 vs. control group; #*p* < 0.05 vs. HG group; ##*p* < 0.01 vs. HG group; $$*p* < 0.01vs. control group for each condition.

Flow detection of apoptosis is divided into four quadrants: Q1-UL (necrotic cells and debris), Q1-UR (late apoptotic cells), Q1-LL (normal cells), Q1-LR (early apoptotic cells); The apoptosis rate is calculated as the sum of the two quadrants of Q1-UR and Q1-LR. From the figure below, we can conclude that the apoptosis rate of the HG group increased compared with the control group (*p* < 0.01), and the apoptosis rate of the HG + CS group had no significant difference compared with the HG group. Compared with the HG + CS group, the HG + DS group decreased the apoptosis rate (*p* < 0.05); compared with the NC-siRNA group; the siRNA-FoxO1 group decreased the apoptosis rate (Figures 13A,B).

#### 3.5.2 YP-SJ formula enhances cardiac autophagy in H9c2 cell

The autophagic outer membrane fuses with lysosomes to form autolysosomes, and the components and inner membranes of lysosomes are degraded by lysosomal hydrolases into proteins, lipids, carbohydrates, nucleic acids, and organelles, which are transported to the cytoplasm reused (Saito et al., 2016). When cells lack the ability of autophagy, intracellular damaged organelles and denatured proteins are not cleared in time, resulting in the destruction of the homeostasis of the internal environment. Excessive autophagy destroys most of the cytosol and organelles, eventually leading to autophagic cell death. Appropriate autophagy can degrade and clear some intracellular non-functional proteins or intracellular organelles and cytoplasmic components to maintain energy metabolism in the intracellular environment. The control group had higher levels of cardiac autophagy (Shirakabe et al., 2016). In this experiment, the number of autophagosomes in the HG group decreased; the number of autophagosomes in the HG + DS group increased compared with the HG + CS group; compared with the NC-siRNA group, siRNA-FoxO1 group autophagosome numbers decreased (Figure 13C).

#### 3.5.3 Immunofluorescence results of H9c2 cells

Using immunofluorescence to detect the changes of FoxO1 expression in H9c2 cells, we found that the expression of FoxO1 in the HG group decreased compared with the control group. Compared with the HG group, there was no significant difference in the expression of FoxO1 between the HG + CS group and the NC-siRNA group. Compared with the HG + CS group, the HG + DS group increased the FoxO1 expression. The siRNA-FoxO1 group decreased the FoxO1 expression compared with the NC-siRNA group (Figure 13D).

#### 3.5.4 YP-SJ formula regulates Beclin1, Atg7, FoxO1, and LC3 II/LC3 I expression in H9c2 cells

**FIGURE 14:**
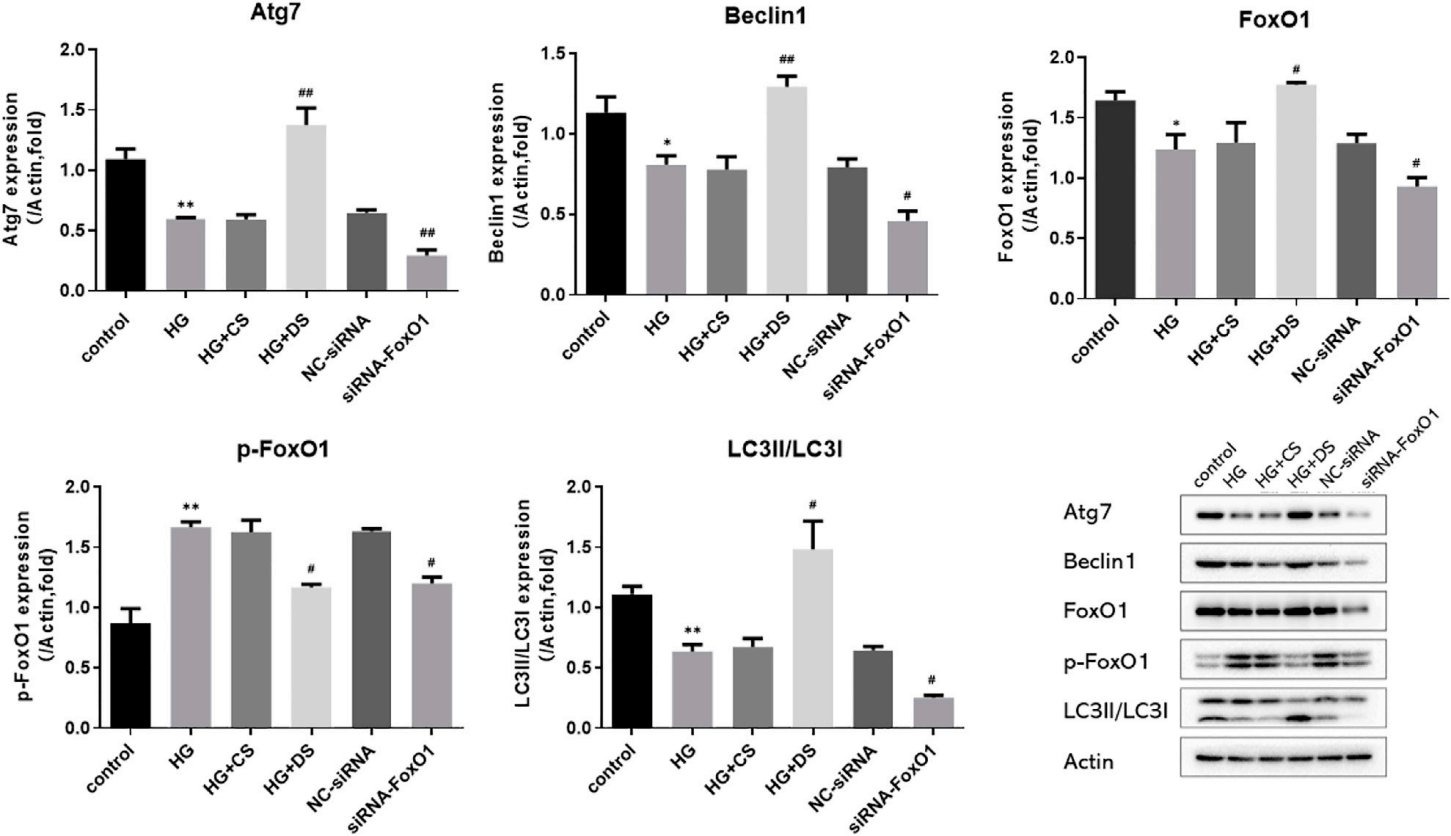
Western blot analysis of Atg7, FoxO1, p-FoxO1, Beclin1, LC3 and Actin protein expression in H9c2 cells. ^*^ *p* < 0.05 vs. control group, ^**^ *p* < 0.01 vs. control group; ^#^ *p* < 0.05 vs. HG group, ^##^ *p* < 0.05 vs. HG group.

Compared with the control group, the expression of Beclin1, Atg7, FoxO1, and LC3 II/LC3 I in the HG group were significantly decreased (*p* < 0.05 and *p* < 0.01), and the expression of p-FoxO1 was significantly increased (*p* < 0.01). Compared with the HG group, the protein levels of Beclin1, Atg7, FoxO1, and LC3 II/LC3 I was significantly increased (*p* < 0.05 and *p* < 0.01), and the expression of p-FoxO1 was significantly decreased (*p* < 0.05) in the HG + DS group. The expression of Atg7, Beclin1, FoxO1, LC3 II/LC3 I, and p-FoxO1 was significantly decreased in the siRNA-FoxO1 group (*p* < 0.05 and *p* < 0.01). (Figure 14).

## 4 Discussions

In the traditional medical practice of various ethnic groups, many plants and minerals have been used for their anti-diabetic effects, especially in T2DM. The compatibility of *Atractylodes Macrocephala* Koidz. And *Citrus aurantium* L. is called Zhizhu Pill. This is from “The Theory of Spleen and Stomach”, written in 1249 AD. They have the effect of promoting food digestion and the metabolism of sugar and lipids (Liu et al., 2008). The combination of *Trichosanthes kirilowii* Maxim. And *Ostreae Concha* is called Gualoumuli powder, from “Synopsis of Prescriptions of the Golden Chamber”, which was written in about 205 AD. Gualoumuli powder is currently used for the treatment of diabetes and has the effect of clearing heat and nourishing yin. The clinical study indicates that Gualoumuli power improves the symptoms of T2DM such as polyphagia, polydipsia, polyuria, blurred vision, and weight loss(Chen and Niu, 1999). *Eupolyphaga sinensis* Walker has been used in TCM for more than 2000 years; it is popular, thought to be good, and can activate the blood. According to the theory of TCM, the pathogenesis underlying DCM is spleen deficiency, yin deficiency, fire exuberance, and blood stasis. YP-SJ formula can significantly improve insulin resistance and reduce the level of IL-6, a vascular inflammatory factor, in type 2 diabetic patients. It also provides a basis for clinical observation on the mechanism of this formula in the prevention and treatment of type 2 diabetic vascular lesions(Zhang and Mao, 2018).

In this study, we demonstrate that YP-SJ formulation can improve myocardial function in DCM rats *in vitro* and *in vivo* by modulating autophagy. However, in view of complex active ingredients of YP-SJ formula and the diversity of therapeutic targets, it is difficult for traditional methods to understand the potential pharmacological mechanism of YP-SJ formula accurately and deeply in the treatment of DCM. Therefore, we employed a novel approach, network pharmacology, to identify active compounds of YP-SJ formulation and predict relevant targets and corresponding mechanisms of action. We constructed and analyzed target networks based on YP-SJ formulation compounds and screened out multiple drug targets. We further revealed through enrichment analysis and functional analysis, emphasizing that YP-SJ formula improves cardiac function by regulating autophagy genes, and found that FoxO1 is a key gene regulating autophagy, and experiments were carried out around FoxO1 as a target.

DCM pathogenesis is generally associated with oxidative stress, microangiopathy, metabolic disorders, and insulin resistance (Erukainure et al., 2021), and we screened the YP-SJ formulation for active ingredients that could treat or alleviate diabetic cardiomyopathy through network pharmacology, in which the phytoestrogen isoflavone may reduce glucose toxicity-induced cardiac mechanical dysfunction and thus has the potential to treat diabetes-related cardiac defects (Hintz and Ren, 2004). Exposure of cardiomyocytes to high glucose leads to functional and structural abnormalities that can be prevented by linoleic acid preconditioning and partially mediated activation of peroxisome proliferator-activated receptor γ (Aloud et al., 2016). Studies have shown that taurine can be involved in the regulation of angiotensin II(Schaffer et al., 2000)and pyruvate dehydrogenase (Hansen, 2001) to exert cardioprotective effects. The present study demonstrated that naringin protected cardiomyocytes from hyperglycemia-induced injury *in vitro* by upregulating KATP channels and inhibiting NF-κB pathway (You et al., 2016). Naringin ameliorated myocardial hypertrophy in diabetic mice, and the mechanism may be related to upregulation of CYP2J3 expression, increase in EETs levels, and activation of PPAR expression (Zhang J. et al., 2018). Quercetin may improve cardiac dysfunction and myocardial fibrosis by attenuating the inflammatory response and dysregulation of glycerophospholipid metabolism in DCM (Jiang et al., 2022). YP-SJ formula may improve cardiac function and alleviate diabetic cardiomyopathy through multiple aspects.

Diabetes can worsen the structure and function of the heart and may lead to heart failure even without coronary atherosclerosis and high blood pressure. Early manifestations are left ventricular diastolic dysfunction, characterized by reduced left ventricular filling speed and late diastolic filling patterns. During disease progression, left ventricular systolic dysfunction occurs with reduced ejection fraction (Murtaza et al., 2019). Structurally, this leads to left ventricular hypertrophy with fibrosis, possibly due to the replacement of apoptotic or necrotic cardiomyocytes by connective tissue (Zamora and Villena, 2019). The development of fibrosis leads to myocardial stiffness and impaired contractility, resulting in systolic and diastolic dysfunction in late DCM due to increased left ventricular wall mass and thickness. This leads to four clinical phenotypes (Mizamtsidi et al., 2016): the first stage is diastolic dysfunction with normal ejection fraction, the second stage is combined systolic and diastolic dysfunction, and the third stage is a non-obstructive microvascular disease or systolic and diastolic dysfunction of coronary atherosclerosis, and the fourth stage is obvious ischemia or infarction that can lead to heart failure. In this study, the myocardium of the DCM model group showed obvious signs of inflammation, necrosis, and fibrosis. At the same time, systolic function indexes FS and EF were decreased, and the diastolic function index E/A was >2, implying impaired myocardial contraction and diastolic function in DCM rats. YP-SJ formula can effectively reduce myocardial inflammation, necrosis, fibrosis, and cardiac damage in DCM rats.

In DCM, IR not only induces a lack of myocardial energy supply but also leads directly to myocardial cell dysfunction and apoptosis. The results of clinical and animal experiments show that YP-SJ formula can effectively reduce blood sugar levels and improve IR. IR interferes with the normal metabolism of cardiomyocytes, causing cardiomyocyte dysfunction and myocardial fibrosis, whereas autophagy can improve myocardial energy metabolism and protect the normal function of myocardial cells. Autophagy has the effect of antagonizing myocardial cell damage caused by T2DM IR (Ceylan et al., 2018). In an animal model of T2DM induced by a HFD combined with STZ, cardiomyocyte autophagy was inhibited (Kobayashi and Liang, 2015; Wu et al., 2018). The results showed that compared with the normal group, the number of myocardial autophagosomes in the DCM rat model was significantly reduced. The same results were also presented in cell experiments. The autophagosomes in the HG group were less than those in the normal group, and the number of autophagosomes increased when the drug-containing serum was added to the high-glucose group. YP-SJ formula effectively increased the level of myocardial autophagy in DCM rats and alleviated myocardial damage. There was an increased number of autophagosomes in the myocardium of the control group because the basal level of autophagy controls the elimination of long-lived proteins and damaged organelles promotes survival and maintains the balance of intracellular metabolism. Under high-glucose conditions, autophagy is inhibited; YP-SJ formula can increase the number of autophagosomes. However, autophagy is a highly dynamic process, and the increase or decrease of autophagosomes is not completely consistent with autophagic activity. Nguyen et al. (Nguyen et al., 2017)reported that the expression of autophagy-related proteins Beclin-1 and LC3II increased with the increase of FFA concentration and induced autophagy in cardiomyocytes. Beclin-1 is an important component of the class III PI3K complex and is involved in the formation of autophagosomes (Kihara et al., 2001). The autophagy marker protein LC3 targets the autophagosome membrane and participates in the formation of autophagosomes. It has two subtypes: LC3Ⅰ and LC3Ⅱ. When autophagy occurs, cytoplasmic LC3 (LC3Ⅰ) is modified by ubiquitin-like processing to form membranous LC3 (LC3Ⅱ) and localized in the entire elongation stage of the autophagosome membrane, and its content is proportional to the number of autophagosomes, which can reflect the level of autophagy activity. In this animal and cell experiment, the level of LC3II/LC3 I protein was increased, and the level of Beclin-1 protein was increased, indicating that autophagy production in cardiomyocytes was promoted.

During IR and hyperinsulinemia, the autophagy activity and transcription levels of genes such as Atg7 are inhibited; this is closely related to transcriptional regulation mediated by the downstream signaling molecule FoxO1 (Duan et al., 2018). Forkhead transcription factors (FoxO) are autophagy-related protein transcription factors that activate ATG genes or autophagy-regulated genes through transcriptional upregulation (Xin et al., 2017). A study shows that knockout of FoxO in insulin-resistant type 2 DCM mice induces autophagy-related gene expression and alters cardiomyocyte injury (Liu et al., 2015). FoxO1 is the main regulator of insulin signal transduction and glucose homeostasis. During starvation, FoxO1 can induce transcription of glucose 6-phosphatase and phosphoenolpyruvate carboxykinase, promote liver gluconeogenesis, and maintain stable blood sugar. However, in conditions of IR and diabetes, due to the lack of regulation of insulin signaling, overactive FoxO1 continues to promote gluconeogenesis in an uncontrolled manner, leading to hyperglycemia and ultimately participating in the occurrence of diabetes and its complications and development (Cheng and White, 2011; O-Sullivan et al., 2015). In cardiomyocytes, FoxO1 activation caused by IR and hyperglycemia participates in myocardial mitochondrial biogenesis and cardiac homeostasis and promotes myocardial remodeling at least in part by increasing the expression of β myosin heavy chain (Qi et al., 2015). Overactivated FoxO1 can also promote the expression of inducible NO synthase in DCM, and further induce the nitrosylation of target proteins such as glyceraldehyde-3-phosphate dehydrogenase and caspase-3, leading to post-hyperglycemia myocardial cell death and myocardial dysfunction (Xin et al., 2017). In terms of autophagy regulation, FoxO positively regulates autophagy and activates a variety of autophagy-related transcription factors, such as Atg7 and Atg12 (Cao et al., 2016). *In vivo* studies in mice have shown that under cell stress conditions such as starvation or myocardial ischemia/reperfusion, it can accelerate the dephosphorylation of FoxO1, and increase the nuclear transcription activity of FoxO1 transcription factors, increase the level of cardiac autophagy, and reduce cardiomyocyte hypertrophy (Sengupta et al., 2009). FoxO1 is a regulatory protein of IR and myocardial autophagy. Quercetin can enhance hypoxia-induced autophagy in pulmonary arterial smooth muscle cells through the FoxO1 pathway (He et al., 2017). Consistent with the abovementioned findings, in this study, we confirmed that protein expression of FoxO1 in the myocardium of the DCM rat model was significantly increased. YP-SJ formula inhibited p-FoxO1 and promoted the transcription of FoxO1 in the nucleus, thereby upregulating the expression of Atg 7. In cell experiments, we used siRNA to silence the FoxO1 gene to further verify the effect of YP-SJ formula drug-containing serum, the protein levels of Atg7, FoxO1, and p-FoxO1 in the siRNA-FoxO1 group were significantly reduced.

## 5 Conclusion

Based on predictions based on network pharmacology, we performed *in vitro* and *in vivo* experiments to further verify the mechanism by which YP-SJ formula effectively improves the further development of DCM. Combined with the existing literature, network pharmacological analysis, and experimental verification, we verified that YP-SJ formula for DCM improves energy metabolism by modulating targets that play a key role in the development of autophagy, presenting multi-component, multi-level, multi-target, multi-pathway, and multi-mechanism characteristics. However, due to the limited experimental conditions, there are still some shortcomings. Using the drug-containing serum for drug research may produce pharmacodynamic results that are closer to the actual conditions *in vivo*. In fact, it is difficult to explain the mechanism of TCM drugs because of its invo metabolic process. Therefore, the pharmacokinetics and tissue distribution of active ingredients of YP-SJ formula *in vivo* will be investigated in subsequent studies. The present study indicated that the effects of YP-SJ formula treating DCM are associated with the promoting autophagy of foxO1, as compared with the effects of foxO1 knockdown on H9c2 cell. However, there are still differences between *in vitro* and *in vivo* experiments. Therefore, the follow-up experiments will use lentivirus transfection or select clinically used drugs such as SGLT-2 inhibitor, DPP-4 Inhibitors, GLP-1 receptor agonists as positive controls to further evaluate the efficacy and mechanisms of treatment with YP-SJ formula *in vivo*.

## Data Availability

The original contributions presented in the study are included in the article/supplementary material, further inquiries can be directed to the corresponding authors.
